# Proceedings of State Sanitary Convention of Pennsylvania

**Published:** 1888-08

**Authors:** 


### BOOK NOTICES.

PROCEEDINGS OF THE STATE SANITARY CONVENTION OF PENNSYLVANIA. Held at Philadelphia, May 12th–14th, 1886.

We are indebted to the Secretary of the State Board of Health for this interesting volume; it contains some very carefully prepared papers upon sanitary matters.

